# Numerical Simulation of Intelligent Fuzzy Closed-Loop Control Method for Radial–Axial Ring Rolling Process of Super-Large Rings

**DOI:** 10.3390/ma15145084

**Published:** 2022-07-21

**Authors:** Ke Zhang, Xiaokai Wang, Lin Hua, Xinghui Han, Xiangjin Ning

**Affiliations:** 1 Hubei Key Laboratory of Advanced Technology for Automotive Components, Wuhan University of Technology, Wuhan 430070, China; zk2013@126.com (K.Z.); hualin@whut.edu.cn (L.H.); nxjnxj2019@163.com (X.N.); 2 Hubei Collaborative Innovation Center for Automotive Components Technology, Wuhan University of Technology, Wuhan 430070, China; 3 School of Automotive Engineering, Wuhan University of Technology, Wuhan 430070, China

**Keywords:** super-large rings, radial–axial ring rolling, offset, circularity, fuzzy control

## Abstract

During the radial–axial ring rolling (RARR) process of super-large rings, abnormal deformation states such as instability and out of circularity often lead to rolling termination and quality fluctuation of ring products. In this work, an intelligent fuzzy closed-loop control method for RARR process of super-large rings is proposed, i.e., the ring’s offset adaptive fuzzy control (ROAFC) based on the regulation of the axial roll’s rotational speed and the ring’s circularity fuzzy control (RCFC) based on the regulation of the mandrel’s feed speed. In addition, a recursive average filtering algorithm is added to smooth the axial roll’s rotational speed and the mandrel’s feed speed according to the actual situation. Using the ABAQUS/Explicit software and its subroutine VUAMP, the intelligent fuzzy controller of the ring’s offset and circularity in the RARR process is designed, and the finite element (FE) model for RARR process of a Φ10 m super-large ring with an integrated intelligent fuzzy control algorithm is established. The variation laws of the ring’s offset and circularity error in the RARR process are studied with regard to different control methods such as conventional planning control (CPC), ROAFC, RCFC, and comprehensive control of ROAFC combined with RCFC (ROAFC + RCFC). The results obtained show that, compared with the CPC, the ring’s offset is reduced by 84.6% and the circularity error is decreased by 51.9% in the RARR process utilizing comprehensive control of ROAFC + RCFC. The research results provide methodological guidance for realizing the intelligent forming of super-large rings.

## 1. Introduction

**Figure 1:**
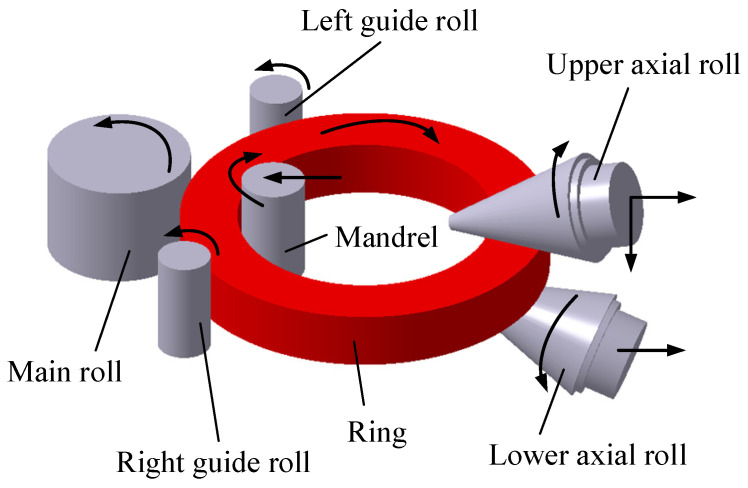
Forming principle of RARR process.

Super-large ring products such as wind power bearing rings, nuclear power supporting rings, and rocket adapters are key structural components used in the field of energy, aviation, aerospace, etc. Radial–axial ring rolling (RARR) is a well-known advanced rotational forming technique to manufacture high-performance super-large seamless rings [1,2,3]. The principle of the RARR process is shown in Figure 1. The main roll rotates around its own axis. The mandrel moves horizontally toward the main roll and passively rotates around its own axis under the friction. The upper axial roll moves downward toward the lower axial roll. Meanwhile, the upper and lower axial rolls make an active rotary motion. The ring rotates due to the active rotation of the main roll and friction; its thickness is reduced by the radial pressure, and its height is reduced by the axial pressure. Consequently, the ring’s diameter is enlarged. A pair of guide rolls, which hug the ring from both sides of the main roll and adjust its position as the ring grows, are used to steer the ring and ensure the ring’s stability during rolling. Synchronously, the upper and lower axial rolls move horizontally backward as the ring is enlarged.

However, the RARR process of a super-large ring takes a long time, and the geometric dimension of the ring changes dramatically. For instance, the rolling process of a Φ10 m aluminum alloy ring of the heavy launch vehicle needs about 30 min. Furthermore, in the final rolling period, the ring’s wall thickness is 3–4 times less than the initial workpiece, the ring’s diameter increases about five times that of the initial workpiece, and the ring’s rotational inertia and stiffness condition change exponentially relative to the initial rolling stage. These features bring challenges for the stability and circularity control of RARR process of super-large rings [3].

To date, much work has been conducted on the plastic deformation laws of RARR process, the effects of different technological parameters such as rolling temperature, rolling speed, and roll dimensions on the ring’s deformation mechanism, and the rolling technology of special material rings. Zhou et al. [4] revealed the effects of axial roll motion and workpiece size on the RARR process, and they studied the RARR process of a Φ9 m super-large steel alloy ring by simulation and experiment. Cleaver et al. [5] established the mechanical models of ring rolling with variable wall thickness and curvature, and they investigated the influence of constraint rolls on temperature distribution and product properties in ring rolling for IN718 ring. Qian et al. [6] established the FE model combining the blank-forging and RARR process of AISI 4140 steel rings, and the evolution laws of strain, grain sizes, and dynamic recrystallization from workpiece to rolled ring during the process were revealed. Lee et al. [7] derived an advanced feasible forming condition for reducing ring spreads and maintaining process stability during the RARR process. Hua et al. [8] established a mathematical model of ring stiffness condition for RARR based on the force method and discussed the influence factors to ring stiffness. Guo et al. [9] proposed constant growth velocity condition (CGVC) of the ring as a design objective of the RARR process variables and developed a mathematical model of the steady forming condition for RARR based on the CGVC. Han et al. [10] put forward an innovative eccentric ring rolling (ERR) method for fabricating eccentric rings, which was verified by simulation and experiment. These studies provide a theoretical guide for realizing a successful and stable RARR process.

FE modeling and numerical simulation have been proven to be a powerful and accurate method to study the plastic deformation behavior of various complicated metal forming processes [4,8,9]. From the forming principle of RARR process, we can know that the process is an extremely complex dynamic forming process with high flexibility. Recently, some significant studies on the RARR process by FE modeling and numerical simulation were reported. For instance, Hua et al. [11] proposed a stiffness model in the RARR process, and its validity was evaluated by numerical simulation. Li et al. [12] established a plastic instability criterion for the ultra-large RARR process with four guide rolls and investigated the influence of guide force on the RARR process of an ultra-large aluminum alloy ring by intelligent FE simulation. Li et al. [13] investigated the influences of the material properties and the forming parameters on the cold ring rolling process through 3D FE numerical simulation. Han et al. [14] established a 3D elastic-plastic FE model for the cylindrical ring rolling process and investigated the comparison between conventional ring rolling and cylindrical ring rolling using this 3D FE model.

In terms of intelligent control of the metal part forming process, Polyblank et al. [15] reviewed the closed-loop control of product properties and revealed its application for controlling errors in geometry and residual stress. Allwood et al. [16] illustrated that uncertainty in various metal forming processes would downgrade product quality and pointed out that the closed-loop control of product properties was essential in metal forming. Havinga et al. [17] used large experimental datasets from an industrial demonstrator process to investigate the feasibility of control of a sheet bending process on the basis of force measurements. Manabe [18] aimed at sheet stamping and tube hydroforming processes, and he designed blank holder force and punch speed fuzzy control algorithm to raise the forming quality of plate parts. Baseri et al. [19] proposed a new fuzzy learning backpropagation (FLBP) algorithm to predict the spring-back using the data generated from experimental observations. As for intelligent control of the RARR process, Liang et al. [20] realized the temperature control of the RARR process of titanium alloy rings by FE simulation. Jenkouk et al. [21] established the FE model of the RARR process coupled with a precompiled industrial control package with all relevant sensors and actuators, which led to more realistic simulation results in comparison to the machine behavior. Peng et al. [22] proposed a new method of adaptive movement control of guide and conical rolls. Li et al. [23] established a new mathematical model to control the guide rolls’ motion during the intelligent simulation of the profiled ring rolling process. However, it seems that there is no study on intelligent control for the RARR process of super-large rings. At the same time, it is of great significance to apply intelligent control to the RARR process of super-large rings to solve the product quality problems caused by the abnormal deformation states of instability and out of circularity. Therefore, it is necessary to establish an intelligent control method for the RARR process of super-large rings to provide guidance for realizing intelligent forming.

In order to effectively describe and control some systems which cannot be modeled mathematically, introducing fuzzy concept is a perfect choice. The fuzzy concept is particularly widespread. For example, the mathematical model is often presented as a differential equation when describing the variation of scalars in the continuous motion of some objects. However, uncertainty of parameters often exists in the process of mathematical modeling. Therefore, in engineering problems, control theory, and decision analysis, there often exist some differential equations with fuzzy sets as parameters, which are called fuzzy differential equations [24,25]. In this study, the fuzzy concept is conveniently used to describe the states of offset and circularity error of the ring during the RARR process, such as large, medium, and small.

In this present work, utilizing the metal forming principle and intelligent control theory, an intelligent fuzzy closed-loop control method for the RARR process of super-large rings is proposed, as well as its FE modeling method with the integrated intelligent fuzzy control algorithm. The ring’s offset adaptive fuzzy control (ROAFC) based on the regulation of the axial roll’s rotational speed is designed. The ring’s circularity fuzzy control (RCFC) based on the regulation of the mandrel’s feed speed is put forward. In addition, a recursive average filtering algorithm is added to smooth the axial roll’s rotational speed and the mandrel’s feed speed. A relevant control program is developed using the ABAQUS/Explicit subroutine VUAMP, and the FE model for RARR process of a Φ10 m super-large ring with integrated intelligent fuzzy control algorithm is established. The variation laws of the ring’s offset and its circularity error during the RARR process are studied with regard to different control methods.

## 2. Materials and Methods

### 2.1. Influence Factors of the Ring’s Offset and Circularity during the RARR Process

**Figure 2:**
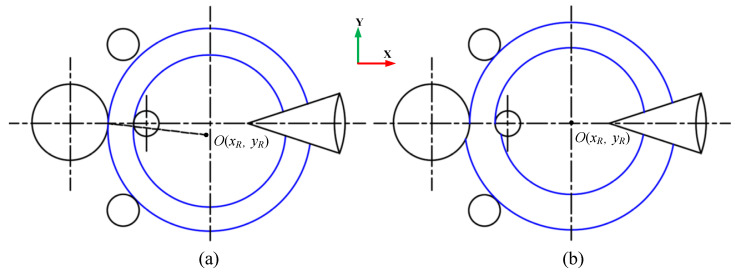
Schematic diagram of (**a**) ring’s offset and (**b**) out of circularity.

During the actual RARR process of super-large rings, when the axial roll’s linear velocity is inconsistent with that of the ring, the ring’s center will deviate from the center line between the main roll and mandrel, and then fluctuate around it, as shown in Figure 2a. On the other hand, the ring’s circularity will become worse when the mandrel’s feed speed is fast and the ring’s growth rate is high, as shown in Figure 2b. Consequently, the ring’s offset and out of circularity can destroy the stability of the RARR process.

#### 2.1.1. Influence of the Axial Roll’s Rotational Speed on the Ring’s Offset

**Figure 3:**
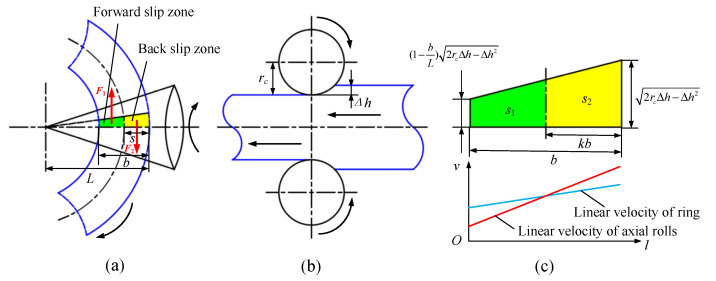
Schematic diagram of interface between axial rolls and ring: (**a**)top view; (**b**)side view; (**c**) projection view and linear velocity distribution diagram.

In fact, the contact type between the axial rolls and ring belongs to the surface contact and the interface is a part of the axial roll’s cone surface during the RARR process. There are both forward slip zones and backward slip zones in this region [26], and the boundary between the forward and backward slip zones is located where the linear velocity of the axial roll’s cone surface is equal to that of the ring’s end face, as shown in Figure 3. In the forward slip zone, the linear velocity of the ring is faster than that of axial rolls, and the ring is affected by the sliding friction force *F*_1_, which hinders its rotation. In the backward slip zone, the linear velocity of the ring is slower than that of axial rolls, and the ring is subjected to the sliding friction force *F*_2_, which propels its rotation. When *F*_1_ ≠ *F*_2_, the ring will deviate from the center line due to unbalance forces; that is, when *F*_1_ > *F*_2_, the hindering effect is stronger than the propelling effect, which will lead to upward offset for the ring. Conversely, the ring will be subjected to downward offset. To simplify the computational model, the interface is projected to obtain approximately a rectangular trapezoid, as shown in Figure 3c. For the convenience of analysis, it is assumed that the boundary between the forward and backward slip zones is a straight line.

In this model, the sliding friction forces *F*_1_ and *F*_2_ are proportional to the areas of the forward and backward slip zone, respectively. Assuming that the area of the forward slip zone is *S*_1_ and the area of the backward slip zone is *S*_2_, this can be respectively expressed as follows:

(1) $$\left\{\begin{array}{l} S_{1}=\frac{b\sqrt{2r_{c}\Delta h-\Delta h^{2}}(\frac{b}{L}k^{2}-2k+2-\frac{b}{L})}{2},\\ S_{2}=\frac{b\sqrt{2r_{c}\Delta h-\Delta h^{2}}(-\frac{b}{L}k^{2}+2k)}{2} \end{array}\right.$$

where *L* is the distance from the vertex of the axial rolls to the contact point between the outside surface of the ring and the axial rolls, *b* is the wall thickness of the ring, *r_c_* is the equivalent radius of the axial rolls at the outer diameter of the ring, Δ*h* is the axial feed amount per revolution, and *k* is defined as a velocity matching coefficient (*k* = *s*/*b*). Let *S*_1_ be equal to *S*_2_; then, a quadratic equation about *k* can be obtained, as expressed by Equation (2).

(2) $$k^{2}-\frac{2L}{b}k+\frac{L}{b}-\frac{1}{2}=0.$$

It is obvious that the variation range of the velocity matching coefficient *k* is from 0 to 1. The solution of Equation (2) is defined as an optimal velocity matching coefficient *k*_0_ and can be expressed by Equation (3).

(3) $$k_{0}=\frac{L}{b}-\frac{\sqrt{L^{2}-bL+\frac{b^{2}}{2}}}{b}.$$

**Figure 4:**
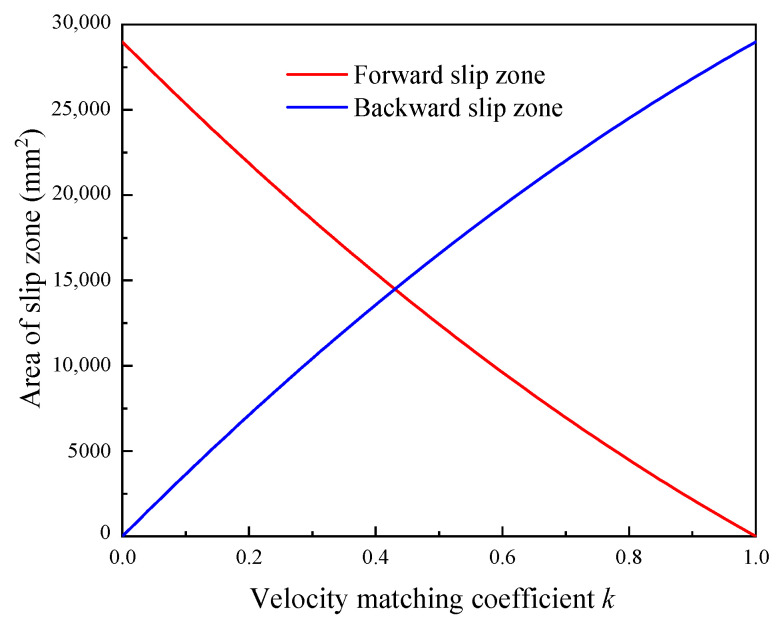
Variation curves of area of slip zone with velocity matching coefficient *k*.

**Figure 5:**
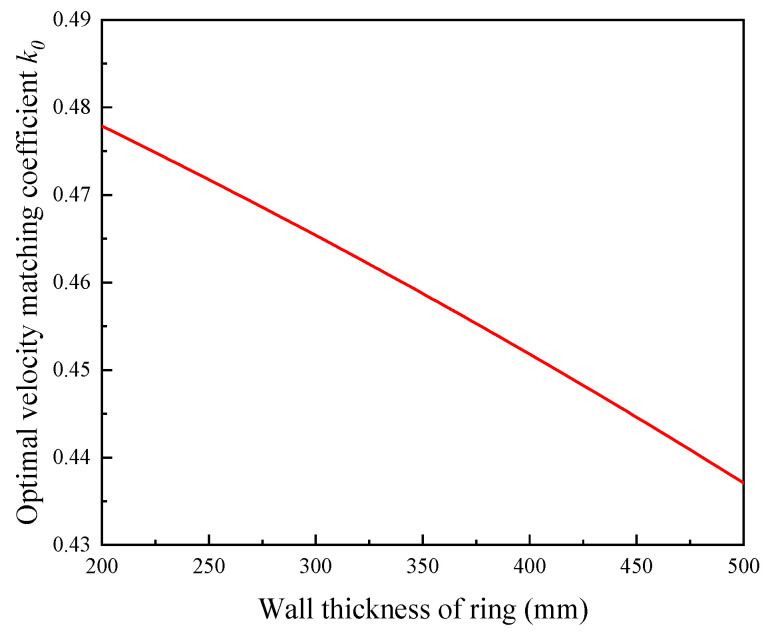
Variation curve of optimal velocity matching coefficient *k*_0_ with ring’s wall thickness.

The variation curves of the areas of forward and backward slip zone with velocity matching coefficient *k* are shown in Figure 4 (*L* = 1228 mm, *b* = 544 mm, *r_c_* = 369 mm, and Δ*h* = 6.4 mm). As can be seen from the Figure 4, with the increase in velocity matching coefficient *k*, the area of the backward slip zone increases continuously from 0 to the maximum. Conversely, the area of the forward slip zone decreases continuously from the maximum to 0. Moreover, when the area of the forward slip zone is equal to the area of the backward slip zone, it can be found that the velocity matching coefficient *k* is about 0.43. The variation curve of the optimal velocity matching coefficient *k*_0_ with the wall thickness of the ring is shown in Figure 5 (*L* = 1228 mm). As can be seen from the Figure 5, with the decrease in wall thickness of the ring, the optimal velocity matching coefficient *k*_0_ increases approximately from 0.437 to 0.477.

As discussed above, it can be known that the value of velocity matching coefficient *k* can affect the areas of forward and backward slip zone, and the value of optimal velocity matching coefficient *k*_0_ changes slightly in the RARR process. On the other hand, the boundary position between the forward and backward slip zones changes accordingly with the change in the axial roll’s rotational speed, which simultaneously represents a change in the value of velocity matching coefficient *k*. Therefore, the rotational speed of the axial rolls can directly affect the kinetic equilibrium of the RARR process, and the reasonable adjustment of the axial roll’s rotational speed can effectively change the sliding friction force *F*_1_ and *F*_2_, so as to suppress the ring’s offset in the RARR process and improve the rolling stability.

#### 2.1.2. Influence of the Mandrel’s Feed Speed on the Ring’s Circularity

**Figure 6:**
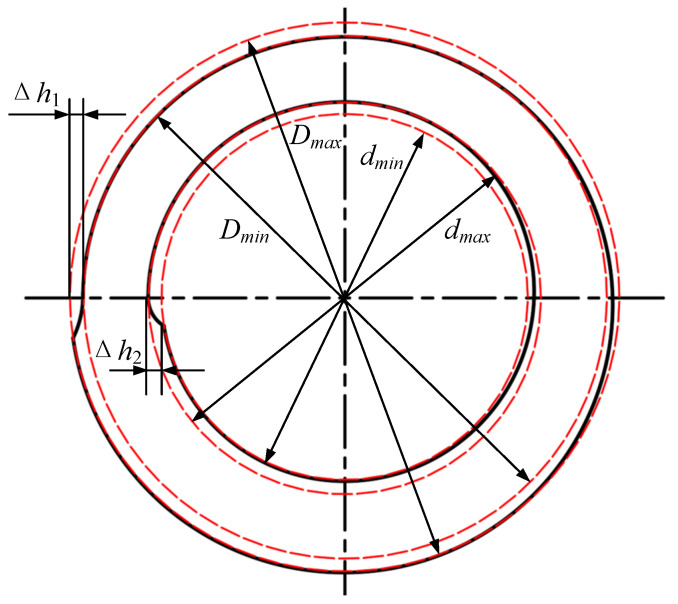
Schematic diagram of the ring’s contour.

Under the condition of the normal ring rolling process, the ring’s contour is composed of an arc line in the deformed zone and a spiral line in the nondeformed zone, as shown in Figure 6. According to the definition that the ring’s circularity error is equal to the difference between the maximum and minimum of the ring’s diameter, the circularity error of the ring’s outer and inner contour *e*_1_ and *e*_2_ can be expressed as Equation (4).

(4) $$\left\{\begin{array}{l} e_{1}=D_{max}-D_{min}\\ e_{2}=d_{max}-d_{min} \end{array}\right..$$

The radial feed amount per revolution Δ*h_r_* distribution to the outside surface is denoted as Δ*h*_1_, while that to the inside surface is denoted as Δ*h*_2_. The relationship between Δ*h_r_* and Δ*h*_1_, Δ*h*_2_ can be expressed as follows [3,6]:

(5) $$\left\{\begin{array}{l} \Delta h_{1}=\frac{\Delta h_{r}(\frac{1}{R_{1}}+\frac{1}{R})}{\frac{1}{R_{1}}+\frac{1}{R_{2}}+\frac{1}{R}-\frac{1}{r}}\\ \Delta h_{2}=\frac{\Delta h_{r}(\frac{1}{R_{2}}-\frac{1}{r})}{\frac{1}{R_{1}}+\frac{1}{R_{2}}+\frac{1}{R}-\frac{1}{r}} \end{array}\right.,$$

where *R*_1_ is the radius of the main roll, *R*_2_ is the radius of the mandrel, and *R* and *r* are the outer and inner radii of the ring, respectively. It is evident that the circularity errors of the ring’s outer and inner contours *e*_1_ and *e*_2_ are equal to the radial feed amount per revolution Δ*h_r_* distribution to the outside surface Δ*h*_1_ and to the inside surface Δ*h*_2_, respectively; that is, *e*_1_ = Δ*h*_1_, *e*_2_ = Δ*h*_2_.

By now, it can be known that the ring’s circularity error is related to the radial feed amount, the roll’s size, and the ring’s size. During the actual RARR process, the size of the rolls and the ring is certain; hence, the ring’s circularity error mainly depends on the radial feed amount per revolution. Therefore, when the circularity error of the ring increases, decreasing the radial feed amount per revolution is beneficial to reduce the circularity error. According to the relationship of the radial feed amount per revolution Δ*h_r_* and the mandrel’s feed speed *v_m_* [27],

(6) $$\Delta h_{r}=\pi D\frac{v_{m}}{v_{R1}},$$

where *D* is the current outside diameter of the ring, and *v_R_*_1_ is the circumferential velocity of the main roll; it can be known from the above equation that the radial feed amount per revolution is proportional to the mandrel’s feed speed. Hence, decreasing the mandrel’s feed speed is beneficial to reduce the circularity error of the ring.

### 2.2. Proposing of the Control Method ROAFC

**Figure 7:**
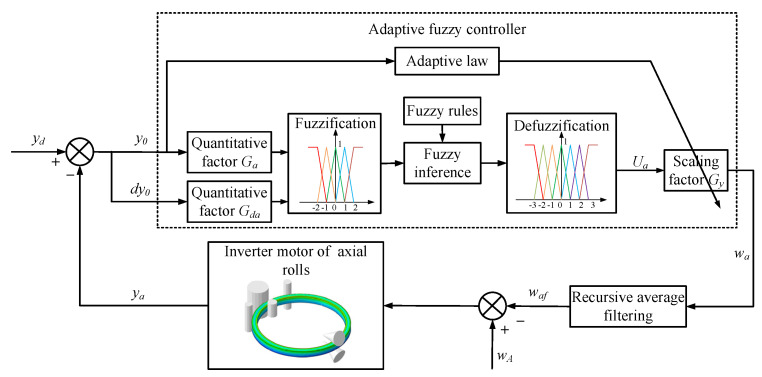
Block diagram of ring’s offset adaptive fuzzy controller.

The framework of the ring’s offset adaptive fuzzy controller was designed as shown in Figure 7. Here, *y_a_* is the actual ring’s center ordinate during the RARR process, and it is sampled as the feedback variable. *y_d_* is the desired value of the ring’s offset, and, in order to control the ring’s offset in a relatively small range, *y_d_* is set to 0. The feedback variable *y_a_* minus the desired value *y_d_* and the input variables of the fuzzy controller are produced as the ring’s offset *y*_0_ and its change rate *dy*_0_. The quantitative factors *G_a_* and *G_da_* are used to normalize *y*_0_ and *dy*_0_ within the ranges of −3 to 3 and −2 to 2, respectively. After the steps of fuzzification, fuzzy inference, and defuzzification, the output variable of the adaptive fuzzy controller *U_a_* is obtained. Another scaling factor *G_y_* is used to tune *U_a_* proportionally according to the actual requirements. The variable after tuning is denoted by *w_a_*, which is the regulating variable of the axial roll’s rotational speed and sums the preset value of the axial roll’s rotational speed *w_A_*. In practice, the radial stiffness of the ring decreases and the rotational inertia of the ring increases exponentially as the RARR process proceeds; thus, the acceleration and deceleration, as well as amplitude limitation, of the axial roll’s rotational speed should be considered. Otherwise, the ring’s offset can be restrained rapidly, but the ring’s circularity will become worse. Therefore, the regulating variable of the axial roll’s rotational speed *w_a_* is processed by the recursive average filtering algorithm and imposed on the inverter motor of the axial rolls in the form of control command; thus, the ring’s offset can be restrained gradually [28].

#### 2.2.1. Design of Adaptive Fuzzy Controller

(1) Fuzzification

**Figure 8:**
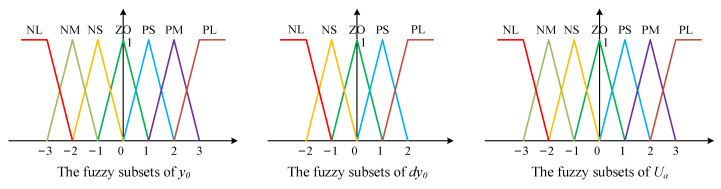
Membership functions of *y*_0_, *dy*_0_, and *U_a_*.

The membership functions can be generalized as bell-, trapezoidal-, and triangular-shaped [29,30]. According to the practical experience and convenience, the triangular and trapezoidal membership functions are used in this research, and the fields of the input variables (*y*_0_, *dy*_0_) and the output variable (*U_a_*) are [−3, 3], [−2, 2], and [−3, 3], respectively. The input variable *y*_0_ is described by seven fuzzy subsets {‘NL’, ‘NM’, ‘NS’, ‘ZO’, ‘PS’, ‘PM’, and ‘PL’}, the input variables *dy*_0_ is described by five fuzzy subsets {‘NL’, ‘NS’, ‘ZO’, ‘PS’, and ‘PL’}, and the output variable *U_a_* is described by seven fuzzy subsets {‘NL’, ‘NM’, ‘NS’, ‘ZO’, ‘PS’, ‘PM’, and ‘PL’}, as shown in Figure 8.

(2) Fuzzy control rules

The fuzzy rules are the most significant part of the fuzzy controller, which have a great influence on the output [31,32]. The rule base of the fuzzy controller is based on the operator’s knowledge and experiences. The most relational words commonly used are ‘if–then’ and ‘also’, whereas ‘and’ is used in a multivariable fuzzy controller [33]. When the ring’s offset occurs, the proficient operators estimate the offset state by means of natural language ‘upward offset’, ‘no offset’, and ‘downward offset’. Then, the operators regulate manually the axial roll’s rotational speed, which can be represented by fuzzy quantities such as ‘large’, ‘modest’, and ‘small’. On the other hand, the existing measurement and control system of RARR mills can also record the data about the operators’ actions and corresponding states of the ring. Therefore, a set of fuzzy control rules can be concluded from the proficient operators or the acquired historical data. By analyzing the proficient operators’ actions and the influence factor of the ring’s offset in the RARR process, it is known that, when the ring is subjected to ‘downward offset’, the axial roll’s rotational speed is reduced by operators; when the ‘upward offset’ state is alleviated, the axial roll’s rotational speed returns to the preset value. Hence, the if–then rules of ROAFC are extracted from the following datasets: (1) if *y*_0_ > 0 and *dy*_0_ > 0, then *U_a_* increases sharply; (2) if *y*_0_ > 0 and *dy*_0_ < 0, then *U_a_* increases slightly; (3) if *y*_0_ < 0 and *dy*_0_ < 0, then *U_a_* decreases sharply; (4) if *y*_0_ < 0 and *dy*_0_ > 0, then *U_a_* decreases slightly. The if–then rules of ROAFC can be described in a fuzzy rules table (Table 1).

(3) Fuzzy inference and defuzzification

**Figure 9:**
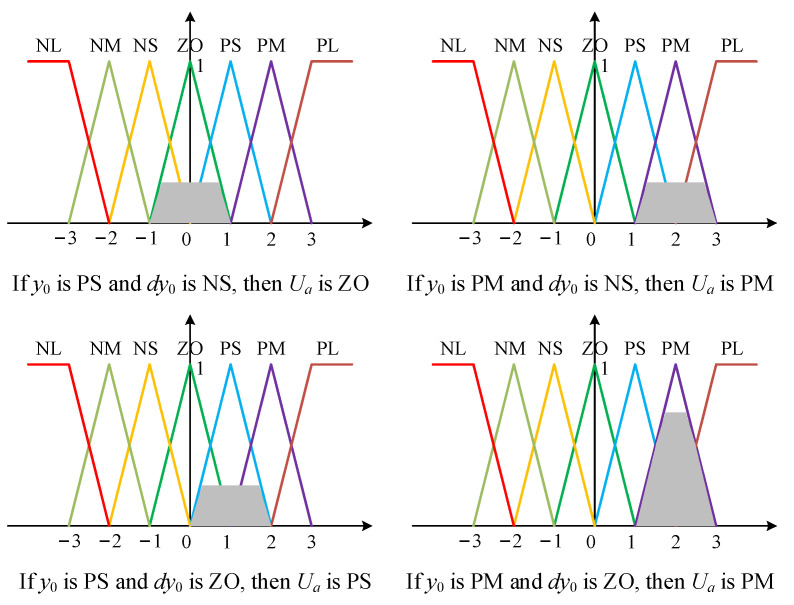
Process of fuzzy inference.

After designing the input and output membership functions and fuzzy control rules, fuzzy inference and defuzzification are needed to calculate the output variable *U_a_*. Four fuzzy control rules were activated, and the Mamdani fuzzy method [34] was employed for fuzzy inference, as shown in Figure 9.

In this research, the weighted mean method [35] was adopted for defuzzification. The output fuzzy subset was defuzzified to obtain the output variable *U_a_*, as expressed by Equation (7). Finally, the regulating quantity of the axial rolls’ rotational speed *w_a_* was obtained by multiplying the scaling factor *G_y_*, as expressed by Equation (8).

(7) $$U_{a}=\frac{\sum_{i=1}^{k}U_{ai}\mu (U_{ai})}{\sum_{i=1}^{k}\mu (U_{ai})},$$

(8) $$w_{a}=G_{y}U_{a},$$

where *k* is the number of the activated fuzzy rules, *U_ai_* is the abscissa of the center of gravity for each fuzzy inference figure, and *μ*(*U_ai_*) is the membership degree of each fuzzy rule.

During the RARR process of super-large rings, when the rolling process proceeds, the ring’s size and rotational inertia increase gradually, and the ring’s offset becomes larger and larger. For the scaling factor *G_y_*, if it keeps a constant value, it will not be able to adapt to the influence for the offset control brought by the large range change of the ring’s size and rotational inertia. Herein, a scaling factor that adaptively changes with the ring’s offset is proposed, which can be mathematically presented as Equation (9).

(9) $$G_{y}=\left\{\begin{array}{l} G_{yb},\left|y_{0}\right|\leq Y_{0}\\ k\left|y_{0}\right|,\left|y_{0}\right|>Y_{0} \end{array}\right.,$$

where *G_yb_* is the basic value of the scaling factor *G_y_*, |*y*_0_| is the absolute value of the ring’s offset *y*_0_, *Y*_0_ is a control threshold of the ring’s offset, and *k* is the adaptive parameter.

#### 2.2.2. Smoothing Filtering Algorithm of the Axial Roll’s Rotational Speed

As mentioned above, the acceleration and deceleration, as well as amplitude limitation, of the axial roll’s rotational speed should be considered, because they change slowly and smoothly in the actual RARR process. This is primarily to alleviate the effect of the dramatic change in the axial roll’s rotational speed on the ring’s circularity and oscillation. In this study, the regulating variable of axial roll’s rotational speed *w_a_* was processed by a recursive average filtering algorithm as follows:

(10) $$w_{af}=\frac{1}{j}\sum_{m=i}^{j+i-1}w_{am},$$

where *w_af_* is the arithmetic average value of the regulating variable of the axial roll’s rotational speed *w_a_* in each sampling period, *j* is the sampling number, *i* is the serial number of the sampling period, and *w_am_* is the value of the regulating variable of the axial roll’s rotational speed *w_a_* arranged by the number *m*.

### 2.3. Proposing of the Control Method RCFC

**Figure 10:**
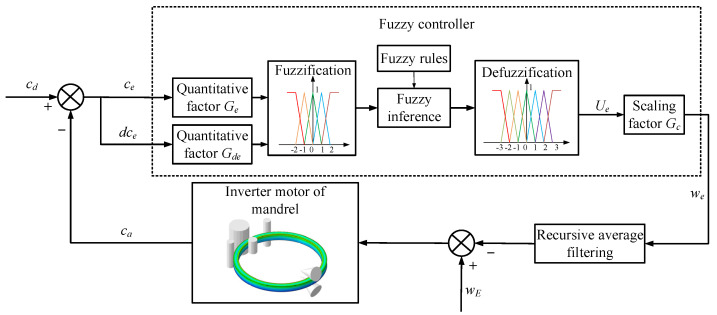
Block diagram of ring’s circularity fuzzy controller.

The framework of the ring’s circularity fuzzy controller was designed as shown in Figure 10. Here, *c_a_* is the actual value of the ring’s circularity error, which is sampled as the feedback variable. *c_d_* is the desired value of the ring’s circularity error, and, in order to control the ring’s circularity error in a relatively small range, *c_d_* is set to 0. The feedback variable *c_a_* minus the desired value *c_d_* and the input variables of the fuzzy controller are generated, i.e., the ring’s circularity error *c_e_* and its change rate *dc_e_*. The quantitative factors *G_e_* and *G_de_* normalize *c_e_* and *dc_e_* within the range of 0 to 6 and −2 to 2. Meanwhile, *U_e_* is the output variable of the fuzzy controller, and the scaling factor *G_c_* is used to adjust *U_e_* proportionally. The variable after adjusting is denoted by *w_e_*, which sums the preset value of the mandrel’s feed speed *w_E_*. Similarly, the summing value of the mandrel’s feed speed *w_e_* was processed by the recursive average filtering algorithm and imposed on the inverter motor of the mandrel in the form of control command. Moreover, the process of fuzzy inference and defuzzification was similar to the way of calculating the output variable *U_a_*, and the process of the smoothing filtering algorithm for the mandrel’s feed speed was analogous to the processing procedure of the regulating variable of axial roll’s rotational speed *w_a_*.

The design of the fuzzy controller is described below.

(1) Fuzzification

**Figure 11:**
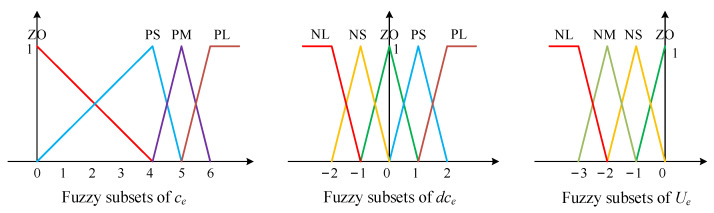
Membership functions of *c_e_*, *dc_e_*, and *U_e_*.

In this study, the fields of the input variables (*c_e_*, *dc_e_*) and the output variable (*U_e_*) were [0, 6], [−2, 2], and [−3, 0], respectively. The input variable *c_e_* is described by four fuzzy subsets {‘ZO’, ‘PS’, ‘PM’, and ‘PL’}, the input variable *dc_e_* is described by five fuzzy subsets {‘NL’, ‘NS’, ‘ZO’, ‘PS’, and ‘PL’}, and the output variable *U_e_* is described by four fuzzy subsets {‘NL’, ‘NM’, ‘NS’, and ‘ZO’}, as shown in Figure 11.

(2) Fuzzy control rules

By analyzing the relationship between the circularity error and the mandrel’s feed speed, it can be known that the mandrel’s feed speed should be reduced when the circularity error of the ring becomes poor. The rules of RCFC can be expressed as a fuzzy rules table (Table 2).

### 2.4. Comprehensive Control Method of ROAFC Combined with RCFC

**Figure 12:**
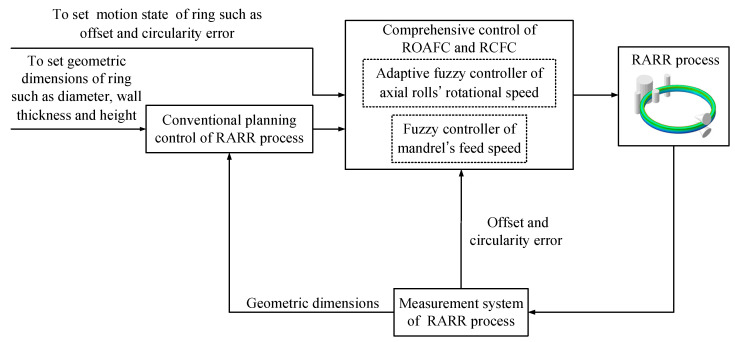
Block diagram of comprehensive control of ROAFC combined with RCFC.

As shown in Figure 12, the comprehensive intelligent fuzzy control of ROAFC combined with RCFC is proposed here, which is complementary to the conventional planning control during the RARR process.

**Figure 13:**
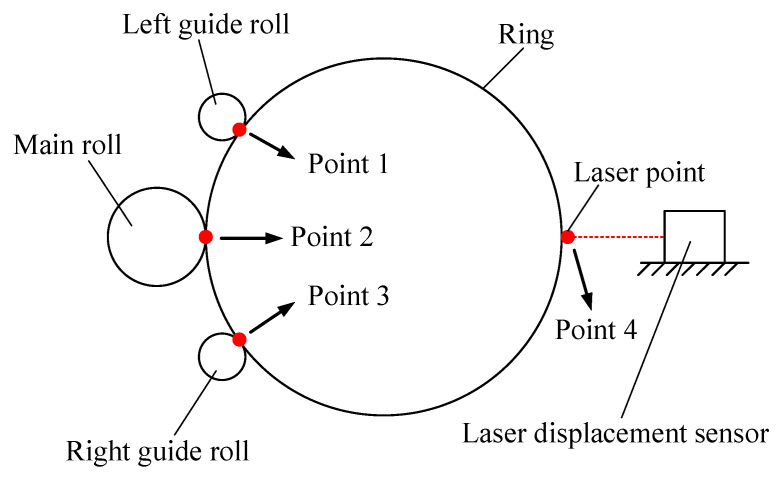
Schematic diagram of measurement method in actual ring rolling process.

It can be seen from Figure 12 that the ring’s offset and circularity error can be obtained by the measurement system of the RARR process. Therefore, it is crucial to understand the principle of the measurement system during the actual RARR process. In this process, the displacements of rolls are obtained by displacement sensors, and the real-time position coordinate of each roll is calculated. Meanwhile, the coordinate of a certain point on the outer wall of the ring is calculated by the noncontact measurement method of the laser displacement sensor, as shown in Figure 13. The radii of the main roll and the mandrel are constant. According to the four position coordinates of the main roll, the left and right guide rolls, and the laser point, the three-point circle fixing method can be used to determine four circles using these four points (point 1, point 2, point 3, and point 4). Then, the center coordinates and radius of each circle can be obtained. Thus, the ring’s offset can be represented by the average value of the ordinates of the four circle centers, and the circularity error can be represented by the difference between the maximum and minimum diameters of the four circles.

### 2.5. Proposing of the Intelligent FE Modeling Method

#### 2.5.1. Establishment of the FE Model with Integrated Intelligent Fuzzy Control Algorithm

**Figure 14:**
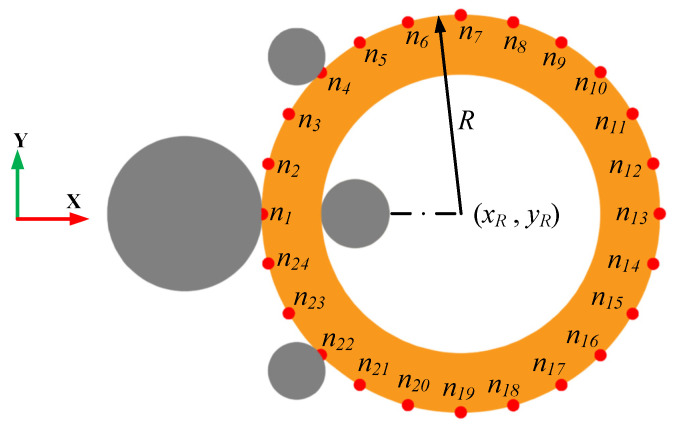
Schematic diagram of measurement of geometric dimension in FE model.

During the actual RARR process, the ring’s offset and circularity are measured and calculated by the roll’s position sensors. This study mainly focused on the controller design and control method for RARR process of super-large rings using the FE simulation method. Therefore, the ABAQUS/Explicit subroutine VUAMP was employed for the secondary development. In order to acquire the information of the ring’s offset and circularity error, several virtual sensors were established in ABAQUS to obtain the coordinates of the nodes on the ring’s outer contour during the simulation [20], and the ring’s offset and circularity error were calculated using the least square circle method [36]. Here, (*x_i_*, *y_i_*) is the coordinate of node *n_i_* on the outer contour of the ring, while (*x_R_*, *y_R_*) and *R* are the center coordinate and radius of the least square circle, respectively, as shown in Figure 14. Additionally, the formula of the least square circle method can be expressed as Equation (11).

(11) $$\left|\right|\sigma {\left|\right|}^{2}=\min{\sum_{i=1}^{n}\left[\sqrt{{\left(x_{i}-x_{R}\right)}^{2}+{\left(y_{i}-y_{R}\right)}^{2}}-R\right]}^{2}.$$

Hence, the ring’s offset and its change rate can be represented by the values *y_R_* and *dy_R_*, respectively. In particular, upward offset and downward offset are expressed as two position statuses of the ring, correspond to *y_R_* > 0 and *y_R_* < 0, respectively. The circularity error *ε_t_* is defined by the difference between the maximum and minimum of the ring’s outer diameter, as expressed by Equation (12).

(12) $$\varepsilon_{t}=e_{max}^{t}-e_{min}^{t},$$

where

(13) $$e^{t}=\sqrt{{\left(x_{i}-x_{R}\right)}^{2}+{\left(y_{i}-y_{R}\right)}^{2}}-R.$$

**Figure 15:**
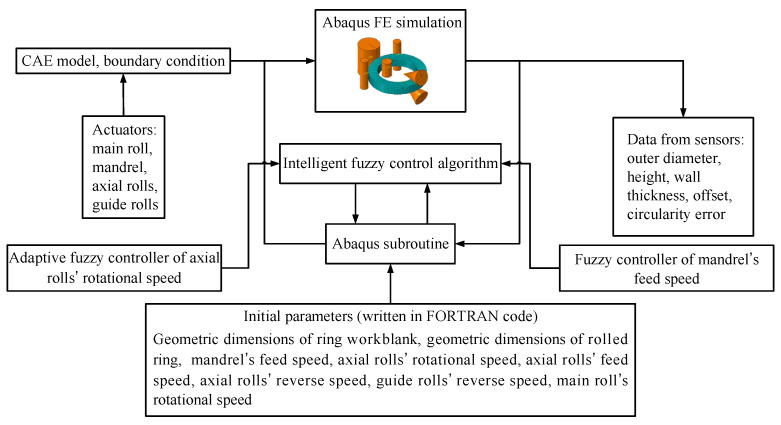
The 3D FE model with integrated intelligent fuzzy control algorithm.

Therefore, the information of the ring’s offset and circularity error is measured by the virtual sensors and is compared with the desired value of the ring’s offset and circularity error, while the signal differences are transferred to the controllers. Subsequently, the actuating signals are output from the controllers and imposed on the corresponding rolls, and then the axial rolls’ rotational speed and the mandrel’ feed speed are regulated. Thus, the intelligent fuzzy closed-loop control method of the ring’s offset and circularity for RARR process was realized during the FE simulation. A 3D coupled thermo-mechanical FE model of a Φ10 m super-large ring with integrated intelligent fuzzy control algorithm was established to simulate the RARR process, as shown in Figure 15.

#### 2.5.2. Material and Technologies of the FE Model

**Figure 16:**
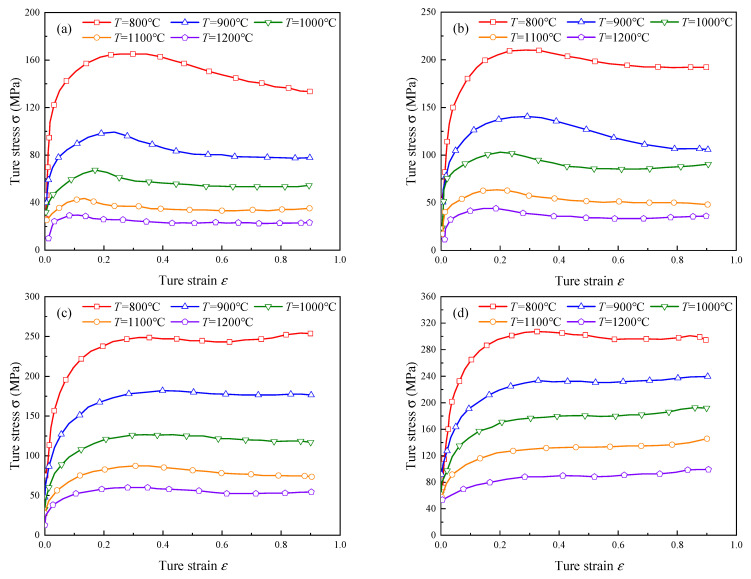
True stress–strain curves of 42CrMo steel at different temperature T and strain rate $\dot{\varepsilon }$: (**a**) $\dot{\varepsilon }$ = 0.01 s^−1^, (**b**) $\dot{\varepsilon }$ = 0.1 s^−1^, (**c**) $\dot{\varepsilon }$ = 1 s^−1^, (**d**) $\dot{\varepsilon }$ = 10 s^−1^.

In the FE model, the used ring material was 42CrMo steel, and its true stress–strain curves [37] at different temperature *T* and strain rate $\dot{\varepsilon }$ are shown in Figure 16. The ring was regarded as deformable body, and the grid type was a coupled thermo-mechanical hexahedron element with eight nodes, C3D8RT. The ring was meshed into 17,280 elements. The rolls were treated as isothermal analytical rigid bodies, and each roll was individually assigned a reference point RP to control its motion. The mass scaling parameter was 400. Six contact pairs were defined between the ring and rolls. The contact pairs were defined as surface-to-surface contact type, and the friction of the contact surfaces adopted the Coulomb friction model. The other key technologies for the FE model of the RARR process and the physical properties, including thermal conductivity, specific heat capacity, and elevated temperature flow behavior, were referenced from Zhou [38]. The main parameters of the FE simulation for RARR process are listed in Table 3. Additionally, under the control methods CPC and ROAFC, the simulation time and CPU time were about 400 s and 15 h, respectively. Under the control methods RCFC and ROAFC + RCFC, the simulation time and CPU time were about 500 s and 21 h, respectively.

### 2.6. FE Model Verification

**Figure 17:**
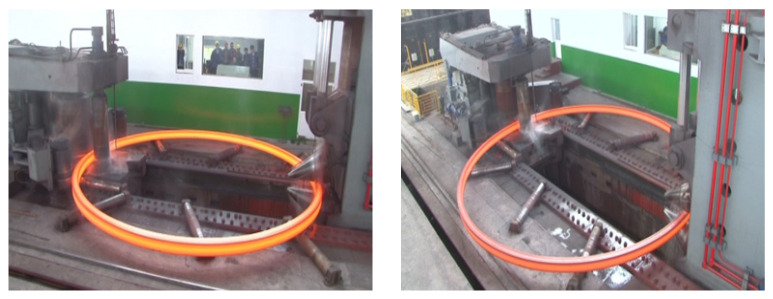
Experiment of RARR process for a super-large ring.

**Figure 18:**
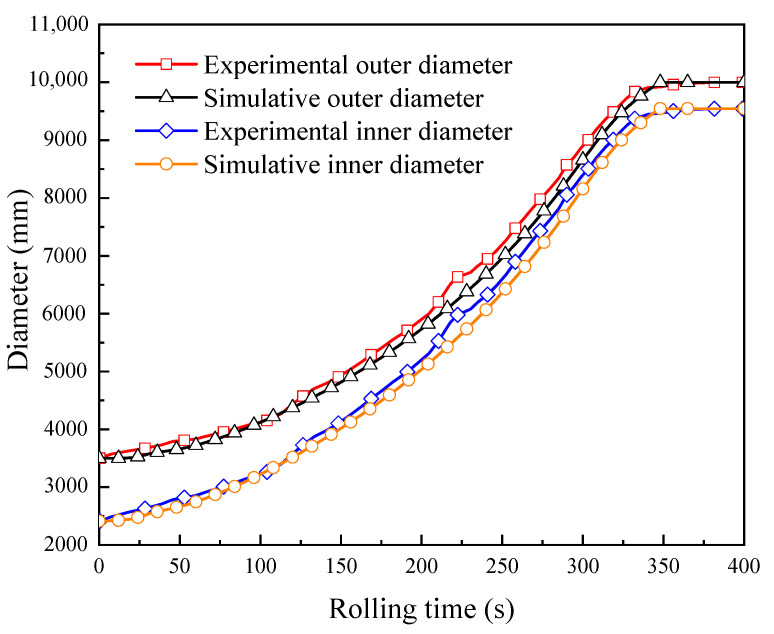
Experimental and simulative variations of the outer diameter and the inner diameter of the ring with rolling time.

**Figure 19:**
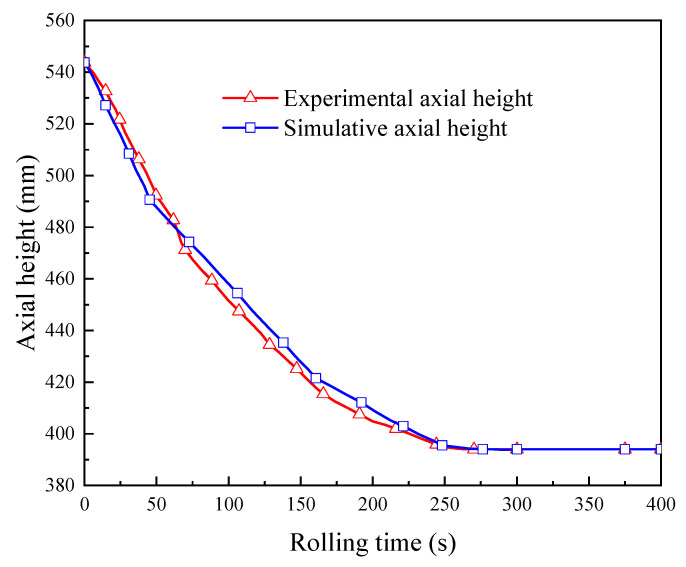
Experimental and simulative variations of the axial height of the ring with rolling time.

The developed FE model was verified by experiment in terms of the variations of the outer diameter, the inner diameter, and the axial height of the ring. The experimental conditions are summarized in Table 3. The ring rolling experimental process is shown in Figure 17. Figure 18 shows the experimental and simulative variations of the outer diameter and the inner diameter of the ring with rolling time, while Figure 19 shows the experimental and simulative variation of the axial height of the ring with rolling time. It can be seen that simulative results were in good agreement with the experimental ones. This indicates that the accuracy of the developed FE model is sufficient for the simulation of proposed control methods.

## 3. Results and Discussion

### 3.1. FE Simulation Results of Forming Process

**Figure 20:**
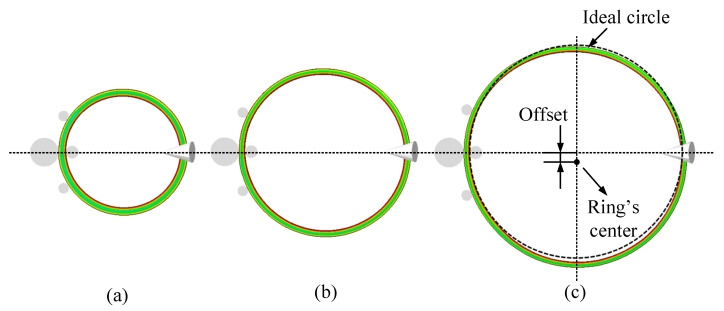
Forming process under the control method of CPC: (**a**) 6 m; (**b**) 8 m; (**c**) 10 m.

**Figure 21:**
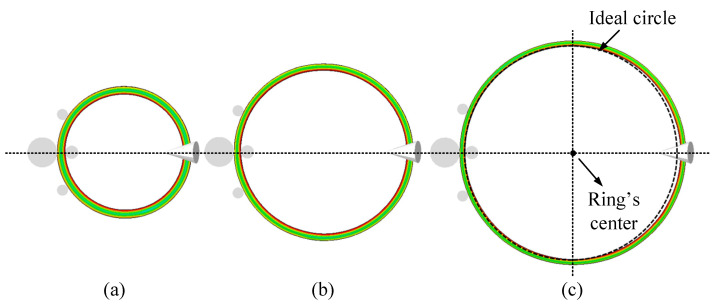
Forming process under the control method of ROAFC + RCFC: (**a**) 6 m; (**b**) 8 m; (**c**) 10 m.

The offset of the ring has a certain impact on its stabilization, which can be used as a standard of the rolling stability during the RARR process of super-large rings. Figure 20 and Figure 21 show the FE simulation results of the ring’s forming process under the control methods of CPC and ROAFC + RCFC, respectively. From the point of view of qualitative description, it can be found from Figure 20c that there was an obvious deviation between the ring’s center and the center line of the mills. Moreover, the overlap extent between the inner contour of the ring and the ideal circle was poor. However, it can be seen from Figure 21c that the inner contour of the ring had a superior overlap extent with the ideal circle, indicating that the ring’s offset was smaller, and the circularity was satisfactory. Therefore, from these two figures, it can be concluded that the comprehensive intelligent fuzzy closed-loop control method of ROAFC + RCFC realized the stable forming process of the ring with better circularity.

### 3.2. Variation Laws of Offset and Axial Roll’s Rotational Speed

**Figure 22:**
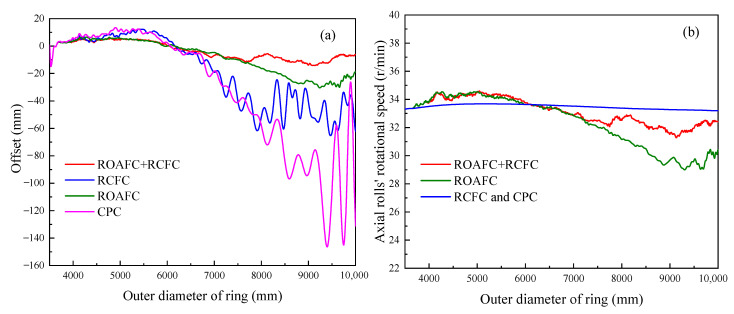
Variation curves under different control methods: (**a**) offset; (**b**) axial rolls’ rotational speed.

Figure 22 shows the variation laws of the ring’s offset and the axial roll’s rotational speed under the different control methods, CPC, ROAFC, RCFC, and ROAFC + RCFC. Under the control method of CPC, it is obvious that the extreme offset was maximal, which could reach about 145 mm. Meanwhile, the variation range of the ring’s offset was the largest at the end of rolling process. Compared with the control method of CPC, the extreme offset was a relatively small value, and the variation range of the ring’s offset was also relatively small at the end of the rolling process under the control method of RCFC. This indicates that the control method of RCFC was beneficial to reduce the ring’s offset. Moreover, the control methods of CPC and RCFC did not involve the adjustment of the axial roll’s rotational speed; thus, the axial roll’s rotational speed remained almost constant throughout the RARR process. Under the control method of ROAFC, the ring’s offset was limited to a small range throughout the RARR process. This illustrates that the control method of ROAFC was effective for decreasing the ring’s offset. Compared with the control method of ROAFC, the ring’s offset was limited to a smaller range during the later stage of the rolling process under the comprehensive control of ROAFC + RCFC, and the extreme offset was about 15 mm. On the other hand, the variation trends of the axial roll’s rotational speed all showed a small increase first, before gradually decreasing, and then remaining within a certain range of fluctuation under the control methods of ROAFC and ROAFC + RCFC. The variation trend was consistent with that of the offset under the corresponding control method, verifying the accuracy of the fuzzy control rules.

### 3.3. Variation Laws of Circularity Error and Mandrel’s Feed Speed

**Figure 23:**
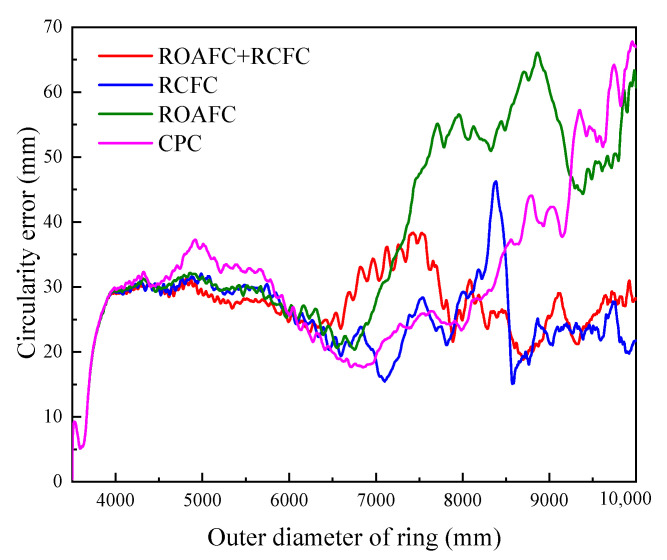
Variation curves of circularity error under different control methods.

**Figure 24:**
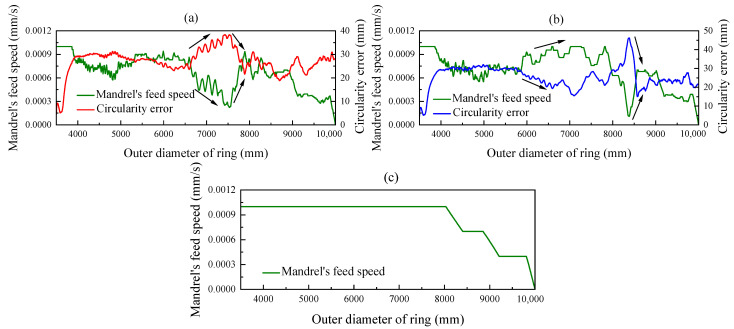
Variation curves of mandrel’s feed speed under different control methods: (**a**) ROAFC + RCFC; (**b**) RCFC; (**c**) CPC and ROAFC.

Figure 23 and Figure 24 show the variation laws of the ring’s circularity error and the mandrel’s feed speed under the different control methods, CPC, ROAFC, RCFC, and ROAFC + RCFC. Under the control methods of CPC and ROAFC, the mandrel’s feed speed remained normal; hence, the ring’s circularity error gradually increased at the end of rolling process and the maximum of the ring’s circularity error exceeded 60 mm. Compared with the control methods of CPC and ROAFC, the ring’s circularity error was kept within a small range during the later stage of the rolling process under the control methods of RCFC and ROAFC + RCFC, not exceeding 35 mm. Furthermore, the variation trend of the mandrel’s feed speed was converse with that of the circularity error in some places under the corresponding control method, as shown in Figure 24a,b, again verifying the accuracy of the fuzzy control rules.

### 3.4. Comprehensive Evaluation

**Figure 25:**
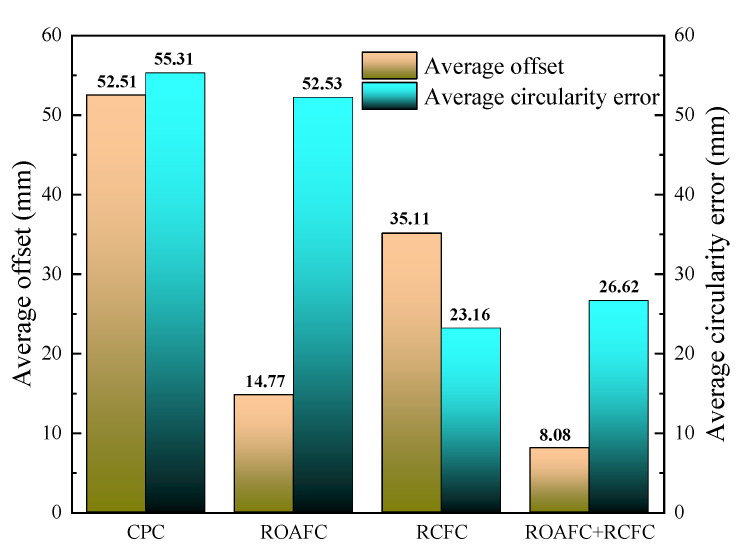
Average offset and circularity error under different control methods.

The ring’s average offset during the RARR process from 6 m to 10 m and the ring’s average circularity error at the end of rolling process from 9 m to 10 m under the different control methods were further analyzed, as shown in Figure 25. It could be obtained that, compared with the control method of CPC, the ring’s average offset and circularity error under the comprehensive control of ROAFC + RCFC were reduced by 84.6% and 51.9%, respectively. The simulation results show that the controller had distinct control effects.

## 4. Conclusions

In this paper, the influence factors of the ring’s offset and circularity error during the RARR process were discussed. The control method of ROAFC for the ring’s offset and RCFC for the ring’s circularity during the RARR process of super-large rings were proposed. On this basis, the fuzzy controllers for the ring’s offset and circularity were designed, and the FE model of the RARR process of a Φ10 m super-large ring with an integrated intelligent fuzzy control algorithm was established. Simulations under the different control methods of CPC, ROAFC, RCFC, and ROAFC + RCFC were carried out. Moreover, the variation laws of the ring’s offset and the corresponding axial roll’s rotational speed, as well as of the ring’s circularity error and the corresponding mandrel’s rotational speed, were analyzed. The results obtained showed that, the maximums of the ring’s offset and circularity error were about 145 mm and 67 mm under the control method of CPC during the RARR process of the Φ10 m super-large ring, respectively. Compared with the control method of CPC, the ring’s average offset and average circularity error were decreased by 84.6% and 51.9%, respectively, under the comprehensive control of ROAFC + RCFC with the recursive average filtering algorithm. In addition, the amplitudes of the change of the ring’s offset and circularity error were gentler. The simulation results verified the feasibility of the intelligent fuzzy closed-loop control method and corresponding controllers. Therefore, it can provide valuable guidance for the intelligent control of the RARR process of super-large rings.

## Figures and Tables

**Table 1:** Fuzzy control rules of ROAFC.

*U_a_*		*dy*_0_				
		NL	NS	ZO	PS	PL
*y*_0_	NL	NL				
	NM	NL		NM		NS
	NS	NM	NS		ZO	
	ZO	NS	ZO			PS
	PS	ZO		PS		PM
	PM	PS	PM		PL	
	PL	PL				

**Table 2:** Fuzzy control rules of RCFC.

*U_e_*		*dc_e_*				
		NL	NS	ZO	PS	PL
*c_e_*	ZO	ZO				
	PS	NS		NM		
	PM	NM		NL		
	PL	NL				

**Table 3:** Main parameters of FE simulation.

Parameters	Value
Outer diameter of the main roll (mm)	1350
Outer diameter of the mandrel (mm)	600
Outer diameter of the guide roll (mm)	500
Cone angle of the axial rolls (°)	35
Outer diameter of the ring blank (mm)	3500
Inner diameter of the ring blank (mm)	2412
Height of the ring blank (mm)	544
Outer diameter of the rolled ring (mm)	10,000
Inner diameter of the rolled ring (mm)	9546
Height of the rolled ring (mm)	394
Initial temperature of the ring blank (°C)	1100
Temperature of the rolls (°C)	80
Temperature of the environment (°C)	20
Heat transmission coefficient (N·s^−1^·mm^−1^·°C^−1^)	10
Heat convection coefficient (N·s^−1^·mm^−1^·°C^−1^)	0.02
Heat radiation coefficient (N·s^−1^·mm^−1^·°C^−4^)	0.7
Friction coefficient	0.35

## Data Availability

Not applicable.
